# Supplementary material for: Covariates of success in quitting smoking: a systematic review of studies from 2008 to 2021 conducted to inform the statistical analyses of quitting outcomes of a hospital-based tobacco dependence treatment service in the United Kingdom
Source: NIHR Open Res. 2023 Oct 20;3:28. Originally published 2023 May 19. [Version 2] doi: 10.3310/nihropenres.13427.2 (PMC10596416; doi:10.3310/nihropenres.13427.2)
Supplement: Peer review history

# Reviewer report: Reviewer response for version 2

**DOI:** 10.3310/nihropenres.14645.r30627

**Published:** 2023-10-23

**Authors:** Ibrahim Ali Kabbash

**Affiliations:** 1 Department of Public Health and Community Medicine, Department Faculty of Medicine, Tanta University, Tanta, Gharbia Governorate, Egypt

The authors responded well to my comments.

Are the rationale for, and objectives of, the Systematic Review clearly stated?

Yes

Is the statistical analysis and its interpretation appropriate?

Not applicable

Are sufficient details of the methods and analysis provided to allow replication by others?

Yes

Are the conclusions drawn adequately supported by the results presented in the review?

Yes

Reviewer Expertise:

community medicine and public health, epidemiology of HIV/AIDS and STIs

I confirm that I have read this submission and believe that I have an appropriate level of expertise to confirm that it is of an acceptable scientific standard.

# Reviewer report: Reviewer response for version 1

**DOI:** 10.3310/nihropenres.14562.r29464

**Published:** 2023-06-27

**Authors:** Ibrahim Ali Kabbash

**Affiliations:** 1 Department of Public Health and Community Medicine, Department Faculty of Medicine, Tanta University, Tanta, Gharbia Governorate, Egypt

The study addressed an important subject; facts affecting success of quitting smoking. The authors used systematic reviewing to collect secondary data from published study and categorized covariables into three categories to explore the determinants of quitting smoking.. The background and rationale of the study were clear and comprehensive. In addition, authors reported in detail the methodology of the study. The discussion and results were satisfactory to give readers insight of the subject. I have a few comments:

 Disagreements were resolved through discussion, with no need to involve a third reviewer. Why did the authors not involve a third reviewer?

- The numbers of studies in figure one showed 95 tests screened out of them 66 were excluded. So, the resulting included in the synthesis should be 29. However, the number mentioned by authors in the figure is 30 studies included in the synthesis and their categorization by general population, mental health and secondary care is 21+6+2=29.
- *“Age*. All studies showed higher odds of quit success with increasing age. Six analyses reported in five papers found no relationship between age and quit success in the UK general population, two studies found no relationship for age in people with mental health conditions, and two studies found no relationship in a secondary care setting.”
  I did not understand how all studies showed a high odd of quitting when the authors mentioned studies showing no relationship. Does it mean that despite heigh odds the risk association was not significant? It needs clarification.

- The authors should identify limitations of the study.

Are the rationale for, and objectives of, the Systematic Review clearly stated?

Yes

Is the statistical analysis and its interpretation appropriate?

Not applicable

Are sufficient details of the methods and analysis provided to allow replication by others?

Yes

Are the conclusions drawn adequately supported by the results presented in the review?

Yes

Reviewer Expertise:

community medicine and public health, epidemiology of HIV/AIDS and STIs

I confirm that I have read this submission and believe that I have an appropriate level of expertise to confirm that it is of an acceptable scientific standard, however I have significant reservations, as outlined above.

# Reviewer report: Reviewer response for version 1

**DOI:** 10.3310/nihropenres.14562.r29469

**Published:** 2023-06-27

**Authors:** Sanjay Agrawal

**Affiliations:** 1 Institute of Lung Health, University Hospitals of Leicester NHS Trust, Leicester, UK

This is an important systematic review that will support the evaluation of hospital in-patient initiated smoking cessation services.

 The authors searched published literature that could indicate factors that affect quit rates and could subsequently be used to evaluate the effectiveness of an individual hospital service or a national evaluation of all hospital services where data is available.

 The systematic review identified 14 'covariates of quitting success' which the authors grouped into themes: demographics, individual health status and healthcare settings, tobacco smoking variables and intervention characteristics, with the authors concluding the 14 covariates be considered for inclusion in datasets and statistical analysis when evaluating quitting outcomes in secondary care in the UK.

 Critique:

 The authors conducted the systematic review following the usual principles and guidelines for conducting systematic reviews. They acknowledged that time constraints and a specific decision to limit their literature search to the certain countries (the UK and Canada) may have reduced information available for the systematic review, indeed only 2 studies from secondary care were identified (it was not possible to see the supplemental tables on the NIHR open research web platform at the time of the review).

 The major critique was not acknowledging the service pathway being implemented for in-patient settings in England, which may in turn have affected the research question and design and outputs of the systematic review. Specifically, the new hospital based services identify people who smoke who are admitted to hospital after which they are seen by in-house tobacco dependency advisors, started on NRT and then have their care transferred to the local community stop smoking service upon hospital discharge, with a 2 week supply of NRT on hospital discharge. These services are still being implemented with a wide range of implementation barriers specific to hospitals for example systematic patient smoking status identification, systematic provision of NRT on admission and discharge, successful transfers of care to community services, employment of  sufficiently trained hospital tobacco dependency advisors, project management support and clinical leadership for service implementation, training of nurses and doctors, IT systems that support case management and service delivery, as well as variation of financial investment and uncertainty of its sustainability. All of these factors will affect quit success as they determine both the 'reach' of the intervention and its 'effectiveness'. Some of these factors may be better identified generically within 'implementation science' or 'change science ' literature rather than specific smoking cessation literature but may have as much of an impact (or greater) than the authors have identified, this certainly seems to be the case from real world feedback from individual hospitals.

 Whilst the information provided in this systematic review is academically sound and very useful, it may be that an additional study be undertaken to look at the wider determinants of quit success beyond those identified using the search strategy for this systematic review.

Are the rationale for, and objectives of, the Systematic Review clearly stated?

Yes

Is the statistical analysis and its interpretation appropriate?

I cannot comment. A qualified statistician is required.

Are sufficient details of the methods and analysis provided to allow replication by others?

Yes

Are the conclusions drawn adequately supported by the results presented in the review?

Partly

Reviewer Expertise:

Tobacco control policy and implementation of tobacco dependency services

I confirm that I have read this submission and believe that I have an appropriate level of expertise to confirm that it is of an acceptable scientific standard.
